# Role of Sodium Bicarbonate Cotransporters in Intracellular pH Regulation and Their Regulatory Mechanisms in Human Submandibular Glands

**DOI:** 10.1371/journal.pone.0138368

**Published:** 2015-09-16

**Authors:** Eun Namkoong, Yong-Hwan Shin, Jun-Seok Bae, Seulki Choi, Minkyoung Kim, Nahyun Kim, Sung-Min Hwang, Kyungpyo Park

**Affiliations:** Department of Physiology, School of Dentistry, Seoul National University and Dental Research Institute, Seoul, 110-749, Korea; University of Szeged, HUNGARY

## Abstract

Sodium bicarbonate cotransporters (NBCs) are involved in the pH regulation of salivary glands. However, the roles and regulatory mechanisms among different NBC isotypes have not been rigorously evaluated. We investigated the roles of two different types of NBCs, electroneutral (NBCn1) and electrogenic NBC (NBCe1), with respect to pH regulation and regulatory mechanisms using human submandibular glands (hSMGs) and HSG cells. Intracellular pH (pH_i_) was measured and the pH_i_ recovery rate from cell acidification induced by an NH_4_Cl pulse was recorded. Subcellular localization and protein phosphorylation were determined using immunohistochemistry and co-immunoprecipitation techniques. We determined that NBCn1 is expressed on the basolateral side of acinar cells and the apical side of duct cells, while NBCe1 is exclusively expressed on the apical membrane of duct cells. The pH_i_ recovery rate in hSMG acinar cells, which only express NBCn1, was not affected by pre-incubation with 5 μM PP2, an Src tyrosine kinase inhibitor. However, in HSG cells, which express both NBCe1 and NBCn1, the pH_i_ recovery rate was inhibited by PP2. The apparent difference in regulatory mechanisms for NBCn1 and NBCe1 was evaluated by artificial overexpression of NBCn1 or NBCe1 in HSG cells, which revealed that the pH_i_ recovery rate was only inhibited by PP2 in cells overexpressing NBCe1. Furthermore, only NBCe1 was significantly phosphorylated and translocated by NH_4_Cl, which was inhibited by PP2. Our results suggest that both NBCn1 and NBCe1 play a role in pH_i_ regulation in hSMG acinar cells, and also that Src kinase does not regulate the activity of NBCn1.

## Introduction

The ability to maintain intracellular pH (pH_i_) homeostasis is critical, and dysregulated pH_i_ is connected with several diseases [1]. Furthermore, pH_i_ can influence various metabolic reactions and vascular functions [2, 3]. There are two major types of proteins that regulate pH_i_, namely, Na^+^-H^+^ exchangers (NHEs) and Na^+^-HCO_3_^-^ cotransporters (NBCs). Moreover, bicarbonate ion (HCO_3_^-^), which functions as a buffer that provides optimal pH, is one of the crucial ions in epithelial cells and fluctuations in the concentration of HCO_3_^-^ in final fluids is associated with several epithelial diseases [4]. HCO_3_^-^ ions in saliva are also protective against enamel erosion under low pH conditions [5]. Thus, bicarbonate transporter is an important protein in the epithelia, especially salivary glands.

NBCs are classified into either electrogenic (NBCe1) or electroneutral (NBCn1) types according to their net transport activity [6]. Human submandibular glands (hSMGs) express two NBC variants, namely NBCe1-B and NBCn1, which were originally cloned as pancreatic and Cl^-^-independent electroneutral NBCs, respectively [7–9]. NBCe1s are further divided into NBCe1-A, -B and -C. NBCe1-A and NBCe1-B are identical except for their N-terminal domains, which are 41 and 85 amino acids in length, respectively [10]. NBCe1-C is the longest of the three NBCe1 variants, and is identical to NBCe1-B except for a unique 61 C-terminal amino acid sequence, which replaces the 46 amino acids of the C-terminus of NBCe1-B [11]. Despite significant research, the role of NBCs in pH regulation as well as the regulatory mechanisms and subcellular localizations of the different NBCs isotypes in hSMG remain elusive.

Protein phosphorylation, a common mechanism of protein regulation, is mediated via the addition of phosphate groups onto serine, threonine, or tyrosine residues. In addition to NBCs, CFTR [12] and neuronal channels such as potassium channels [13] and NMDA receptors [14] are controlled by Src family tyrosine kinases (SFK). In renal epithelial cells, non-receptor tyrosine kinase proline-rich tyrosine kinase 2 (Pyk2) increases the activity of NBCe1 by autophosphorylation and interactions with Pyk2-Src family kinases [15]. However, the role and regulatory mechanisms of the specific isotypes of NBCe1 and NBCn1 in hSMG remain poorly understood. Interestingly, we found that the Src kinase inhibitor PP2 alters pH_i_ regulation in an HSG cell line originating from hSMG duct cells.

In the present study, we studied the expression of NBCn1 and NBCe1-B in hSMG and HSG cells. We also examined whether NBCn1 in hSMG cells plays a role in pH_i_ regulation and investigated its regulatory mechanism via tyrosine phosphorylation compared with NBCe1-B.

## Materials and Methods

### Source of Human Submandibular glands (hSMGs)

Human submandibular glands (hSMGs) were obtained from patients who underwent resection of their submandibular gland as part of their treatment for oral tumors. The patient group included both males and females ranging from 37 to 82 years of age. Tissues were kept in cold physiological saline while transporting the dissected gland from the hospital to the laboratory for analysis. Some of the tissues were fixed with 4% paraformaldehyde for immunohistochemistry studies while the remainder were prepared for physiological experiments. All patients gave written informed consent for participation in this study. The collection and use of human tissue was performed according to ethical guidelines and the study protocol was approved by the Institutional Review Board of Seoul National University Dental Hospital (CRI11023G).

### hSMG acinar cell preparation

hSMG acinar cells were prepared as described previously [16]. Briefly, after trimming fat and connective tissues, tissues were minced with scissors in an ice-cold Ca^2+^-free incubation solution containing 130 mM NaCl, 4.5 mM KCl, 1 mM NaH_2_PO_4_·2H_2_O, 1 mM MgCl_2_, 10 mM D-glucose, and 10 mM HEPES at pH7.4. The minced tissue was then incubated for 60 min at 37°C in a Ca^2+^-free incubation solution containing 2 mg/mL trypsin inhibitor (Sigma, St. Louis, MO, USA), 0.04 mg/mL collagenase P (Worthington, Lakewood, UK), and 1% BSA. During the incubation period, the tissue was mechanically dissociated by repeated pipetting with different sizes of 1 mL pipet tips at 20 min intervals. The isolated acinar cells and cell clusters were then filtered through a 200 μm nylon mesh to remove large debris and harvested by centrifugation.

### Cell culture

HSG cells, which were isolated from human submandibular gland intercalated duct cells [17], were cultured in Dulbecco’s modified Eagle’s medium (Welgene, Republic of Korea) supplemented with 10% fetal bovine serum (Welgene) and 1% penicillin/streptomycin (Gibco, Carlsbad, CA, USA) at 37°C in a 5% CO_2_ atmosphere.

### Reverse Transcription-Polymerase Chain Reaction (RT-PCR)

Total RNA was extracted from hSMG acinar cells obtained from the hSMG acinar cell preparation, whole hSMG, and HSG cells using Trizol (Invitrogen, Carlsbad, CA, USA). Reverse transcription reactions were performed using 1 μg total RNA to generate cDNA (Invitrogen). PCR was performed with 1 μL of cDNA and specific primers (Table 1). The cycling parameters were as follows: 32 cycles of denaturation at 95°C for 30s, annealing for 30s, and extension at 72°C for 30s, followed by a final extension at 72°C for 10 min. Products from RT-PCR reactions were sequenced to confirm their identity.

**Table 1:** List of DNA primers sequences designed for RT-PCR.

Target gene	Forward primers	Reverse primers	Length	GenBank no.
NBCe1-A	ACCTTGGGGAGAGAGGAAGA	TCCTTCCACTCCATCTCCTG	214 bp	NM_003759.3
NBCe1-B/C	TGGAGGATGAAGCTGTCCTG	TGCAGCAGGAGAGATGAGAG	266 bp	NM_001134742.1 / NM_001098484.2
NBCe1-A/B/C	AGCATGACCTCAGCTTCCTG	CAGCATGATGTGTGGCGTTC	253 bp	NM_003759.3 / NM_001098484.2
NBCe1-A/B/C	AGCATGACCTCAGCTTCCTG	CAGCATGATGTGTGGCGTTC	156 bp	NM_001134742.1
NBCn1	CCCAGTCTGCTCCTGGAAAC	ACCCTGTAAGGAGGACAGCA	234 bp	NM_003615.4
AQP5	TCCATTGGCCTGTCTGTCAC	CACTCAGGCTCAGGGAGTTG	211 bp	NM_001651.3
GAPDH	CATCACTGCCACCCAGAAGA	GTCAAAGGTGGAGGAGTGGG	349 bp	NM_001289745.1

### pH_i_ measurements

Intracellular pH (pH_i_) measurements were performed as described previously [18]. HSG cells were loaded with 2 μM BCECF-AM (Molecular Probes, Eugene, OR, USA) for 30 min at 37°C in DMEM medium (10% FBS and 1% penicillin/streptomycin) and washed twice with PBS. Isolated hSMG acinar cells were incubated with 2 μM BCECF-AM for 30 min at room temperature in normal HEPES solution with 0.2% BSA and washed twice with normal HEPES. The standard HCO_3_^-^-buffered solution consisted of 10 mM D-glucose, 10 mM HEPES, 115 mM NaCl, 5 mM KCl, 1 mM MgCl_2_, 1 mM CaCl_2_, and 25 mM NaHCO_3_, and equilibrated with 95% O_2_ + 5% CO_2_ gas. For intracellular acidification, cells were perfused with 20 mM NH_4_Cl in a Na^+^-free bath solution. In Na^+^-free HCO_3_^-^-buffered solutions, NaCl, and/or NaHCO_3_ were replaced with equimolar concentrations of N-methyl- D-glucamine Cl and choline-HCO_3_^-^, respectively. All solutions were adjusted to pH 7.4 at 37°C. pH_i_ recordings were obtained using a MetaFlour imaging system and BCECF was excited at 440 and 490 nm with an emission wavelength of 530 nm. The resulting excitation/emission ratios were converted into pH values using a nigericin-based calibration technique. S1 Fig shows calibration curves. Specifically, cells were perfused with calibration solutions containing 10 mM NaCl, 130 mM KCl, 0.8 mM MgCl_2_, 20 mM HEPES, and 0.005 mM nigericin corresponding to pH 6.2, 6.6, 7.0, 7.4, and 7.8.

### Immunofluorescence

hSMG tissues were cut into small pieces, fixed with 4% paraformaldehyde for at least 48 hours at 4°C, and embedded in paraffin. After microdissecting the block into 10 μm thick sections, the tissues were deparaffinized with Histo-Clear II and rehydrated with a graded series of ethanol (100%, 90%, 80%, and 70%). Sections were incubated in pepsin antigen retrieval solution for 15 minutes at 37°C and permeabilized in PBS containing 0.1% Triton X-100. After permeabilization, the tissues were covered with a blocking solution consisting of PBS with 20% normal donkey serum for 1 h at room temperature. Next, the cells were stained with either rabbit anti-NBCe1 (ab78326, Abcam) or rabbit anti-NBCn1 (ab82335, Abcam) followed by Alexa Flour 468 donkey anti-rabbit IgG (Invitrogen) at a dilution of 1:400 to detect NBCe1 and NBCn1, respectively. Finally, tissues were mounted with Vectashield mounting medium (Vector Laboratories) and visualized using a laser scanning confocal microscope (LSM 700, Carl Zeiss).

HSG cells were grown on cover-glass bottom dishes, fixed in ice-cold methanol for 15 minutes at -20°C, and then washed in ice-cold PBS three times for 5 minutes. The samples were then incubated in PBS containing 1% Triton X-100 for 10 minutes at room temperature, followed by washing in PBS three times and re-incubating in blocking solution consisting of PBS with 10% normal donkey serum for 1 hour at room temperature. The primary and secondary antibodies as well as methods for detection and visualization were the same as those described for hSMG tissues.

### Plasmid Construction, Transfection, and Co-immunoprecipitation

NBCe1-B was cloned in pFlag-CMV-2 vector for expression in HSG cells. The NBCn1 construct in pcDNA3.1 was a gift from Dr. Jeong Hee Hong and Dr. Shmuel Muallem. pFlag-CMV-2-NBCe1-B, pcDNA3.1-NBCn1, and empty vectors were transfected into HSG cells using Lipofectamine 2000 (Invitrogen). Co-immunoprecipitation experiments were performed as described previously [18].

### Reagents

5-(*N*-ethyl-*N*-isopropyl) amiloride (EIPA) and 4,4-diisothiocyanostilbene-2,2-disulfonic acid (DIDS) were obtained from Sigma-Aldrich.

### Statistical analysis

Data are presented as the mean ± SEM and n is the number of experimental repeats with different hSMG sample preparations and HSG cultures. Differences between means were evaluated by ANOVA followed by post-hoc test for determining statistical analysis where appropriate. Statistical significance of experiments with multiple comparisons was assessed by analysis of variance; *p* < 0.05 was considered significant. *, *p* < 0.05; **, *p* < 0.01; ***, *p* < 0.001.

## Results

### Expression of NBCe1 and NBCn1 in human submandibular glands (hSMGs) and an HSG cell line

**Fig 1:**
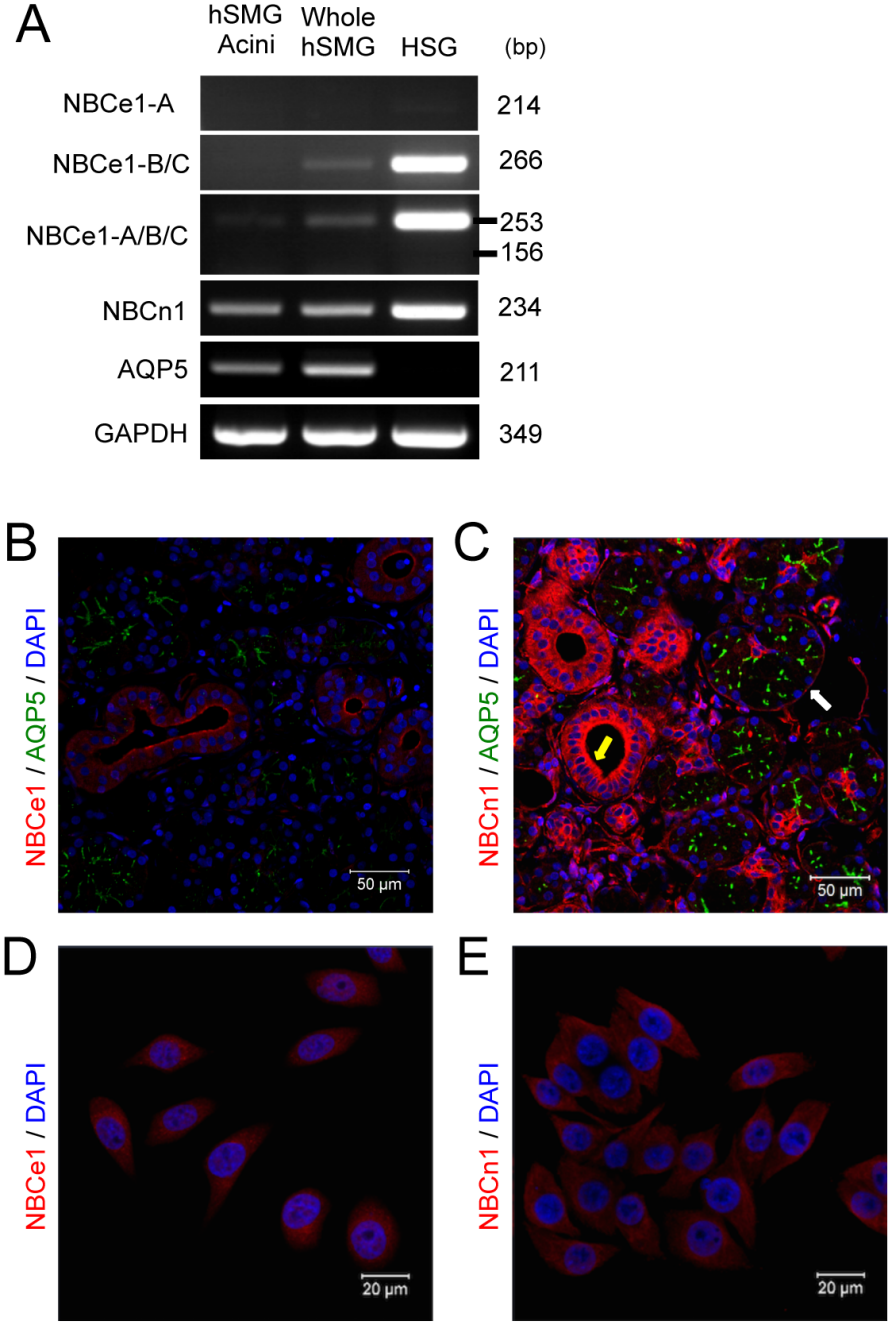
NBCe1 and NBCn1 are expressed in human submandibular gland (hSMG) and HSG cells. (A) NBCe1 and NBCn1 mRNA transcripts in hSMG and HSG cells. Aquaporin 5 (AQP5) was used as a marker for acinar cells. (B and C) hSMG tissue sections were stained with NBCe1, NBCn1, and AQP5 antibodies. (Bar = 50 μm). AQP5 was used as a marker for acinar cells. White and yellow arrows indicate acinar cells and duct cells, respectively. NBCe1-B is expressed in human submandibular gland (hSMG) duct cells, whereas NBCn1 is expressed in acinar (white arrow) and duct cells (yellow arrow). (D and E) HSG cells were stained with antibodies for NBCe1 and NBCn1. (Bar = 20 μm).

We used RT-PCR to investigate the expression of various isotypes of sodium bicarbonate cotransporters (NBCs) in human submandibular glands (hSMGs) and HSG cells originating from hSMG ducts. Three types of primers were designed for NBCe1, one to detect NBCe1-A only at 214 bp, another to detect both NBCe1-B and -C at 266 bp, and the other to detect each of the NBCe1 isoforms (NBCe1-A/B/C, Table 1) at 253 bp and 156 bp corresponding to NBCe1-A/B and NBCe1-C, respectively. The NBCe1 mRNA transcripts, except NBCe1-A, were expressed in whole hSMGs and HSG cell lines, but appeared as a faint band in hSMG acini (Fig 1A). With respect to the NBCe1-A/B/C primer, only the 253 bp product was detected, indicating that only NBCe1-B, but not NBCe1-C, was expressed at the mRNA level in hSMG duct cells and HSG cells. NBCe1-B/C mRNA transcripts were also detected, indicating that NBCe1-B is expressed at the mRNA level in both hSMG ducts and HSG cells. These findings were consistent with the results of our previous study [18]. On the other hand, NBCn1 mRNA transcripts were detected in hSMG acinar cells, whole hSMGs, and the HSG cell line. In these assays, AQP5 was used as an acinar cell marker [19].

To confirm the protein expression and localization of NBCe1-B and NBCn1 in hSMGs and HSG cells, immunostaining was performed with NBCe1 and NBCn1 antibodies. NBCe1 was strongly expressed on the apical side of all hSMG duct cells (Fig 1B), whereas NBCn1 was expressed on the basal side of acinar cells (white arrows, Fig 1C) and on the lateral and possibly basal membrane of duct cells (yellow arrow, Fig 1C). NBCe1 and NBCn1 were diffusely located in both the cytosol and membrane in HSG cells (Fig 1D and 1E). Taken together, these data demonstrated that NBCe1-B is expressed in hSMG ducts and HSG cells, whereas NBCn1 is expressed in the acinar and duct cells of hSMGs and HSG cells.

### Intracellular pH (pH_i_) regulation and the effect of Src tyrosine kinase in hSMG acini and HSG cells

**Fig 2:**
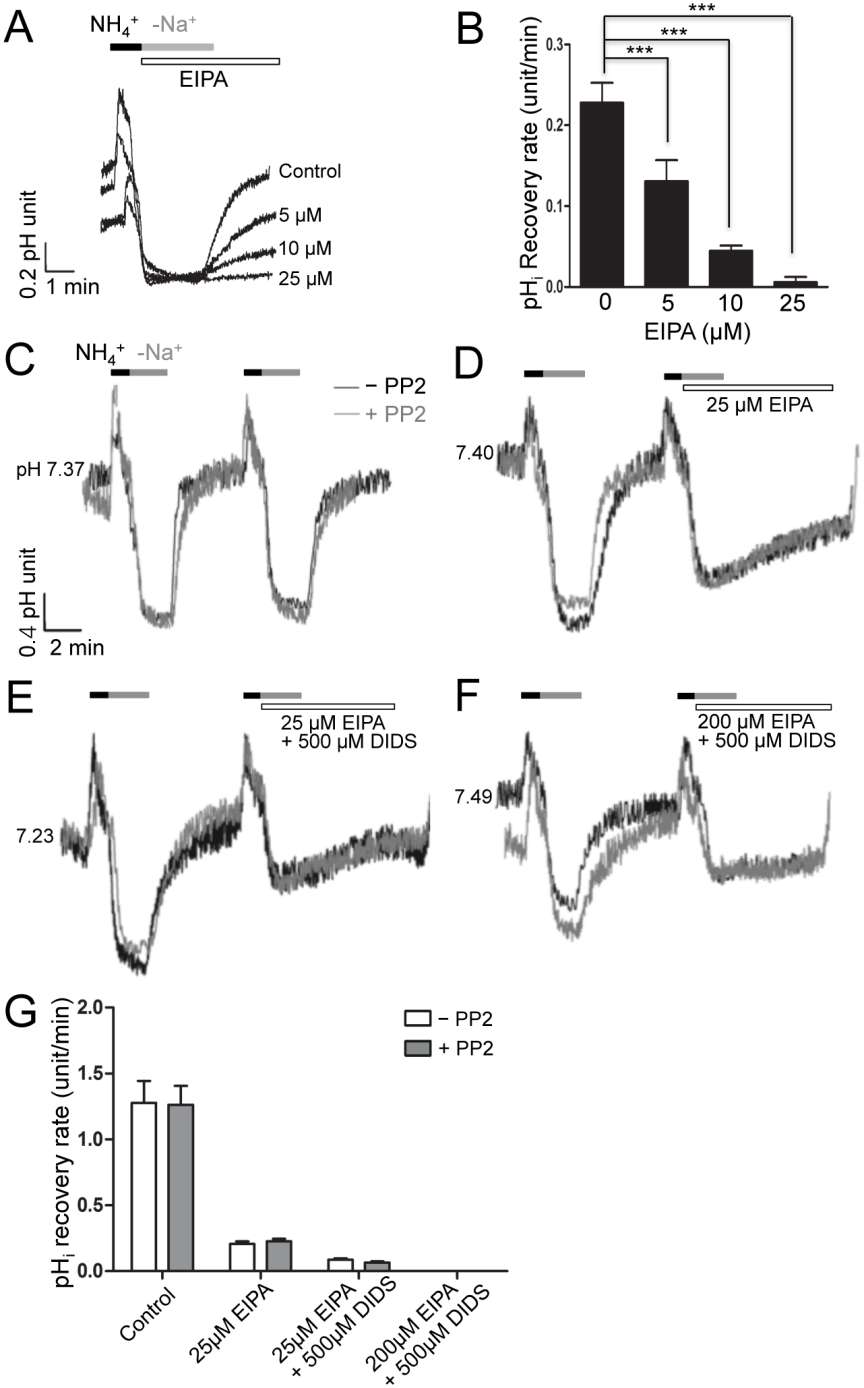
Src tyrosine kinase does not affect pH_i_ recovery of hSMG acinar cells. (A and B) The intracellular pH recovery patterns of hSMG acinar cells in the absence or presence of several concentrations of EIPA in HEPES-buffered solution (HBS) were measured and the pH_i_ recovery rates were summarized. (C-F) The pH_i_ recovery patterns of hSMG acinar cells following an NH_4_^+^-pulse (blank bar) were recorded in a bicarbonate-buffered bath solution (BBS). The cells were pretreated for 20 min with 5 μM PP2, a Src tyrosine kinase inhibitor (grey trace) or incubated in normal BBS (black trace). The effects of treatment with EIPA and DIDS are shown using horizontal bars. (G) Summary of pH_i_ recovery rates. The data are presented as the mean ± S.E.

To evaluate the activity of NBCs on pH_i_ recovery, Na^+^-H^+^ exchanger (NHE) activity should be blocked, as NHEs also regulate intracellular pH. We first examined NHE activity on Na^+^-dependent pH_i_ recovery from cell acidification induced by an NH_4_^+^ pulse in hSMG acinar cells in a HEPES buffered solution (Fig 2A). The pH_i_ recovery rate mediated by NHE was inhibited by 5-(*N*-ethyl-*N*-isopropyl) amiloride (EIPA) in a concentration dependent manner. In addition, the pH_i_ recovery rate of 0.228 ± 0.010 pH units/min (n = 6) in resting states was completely inhibited by 25 μM EIPA, a specific NHE inhibitor (n = 6, Fig 2B).

We measured pH_i_ in an HCO_3_^-^-buffered solution (BBS) to investigate NBCs activity. The pH_i_ of the unstimulated cell was 7.36 ± 0.05 (n = 16) in BBS. When the cell was exposed to NH_4_Cl, the pH_i_ was increased to 9.16 ± 0.13 (n = 16), and then the cell was acidified to pH 6.31 ± 0.04 (n = 16). As shown in Fig 2C, pre-incubation of cells for 20 min with 5 μM PP2, a Src tyrosine kinase inhibitor, had little effect on pH_i_ recovery in hSMG acinar cells in BBS. Further, the pH_i_ recovery rate (grey lines, 1.263 ± 0.142 pH units/min, n = 12) was not significantly different from that of control cells (black lines, 1.277 ± 0.166 pH units/min, n = 12). Continued recovery of pH_i_ was observed in the presence of 25 μM EIPA (0.207 ± 0.018 pH units/min, n = 9, Fig 2D), indicating that remnant pH_i_ recovery was mediated by NBCn1. In addition, the pH_i_ recovery rate of PP2 pretreated hSMG acinar cells (grey lines, 0.228 ± 0.018 pH units/min, n = 8) was not significantly different from control cells. The pH_i_ recovery mediated by NBCn1 was further inhibited by 25 μM EIPA and 4,4-diisothiocyanostilbene-2,2-disulfonic acid (DIDS) (0.087 ± 0.011 pH units/min, n = 8, Fig 2E), and was completely blocked in the presence of high concentrations of EIPA and DIDS (n = 8, Fig 2F). Together, these data suggested that NBCn1 regulates intracellular pH in hSMG acinar cells, but is not affected by Src tyrosine kinase. Fig 2G summarizes the results of these experiments.

**Fig 3:**
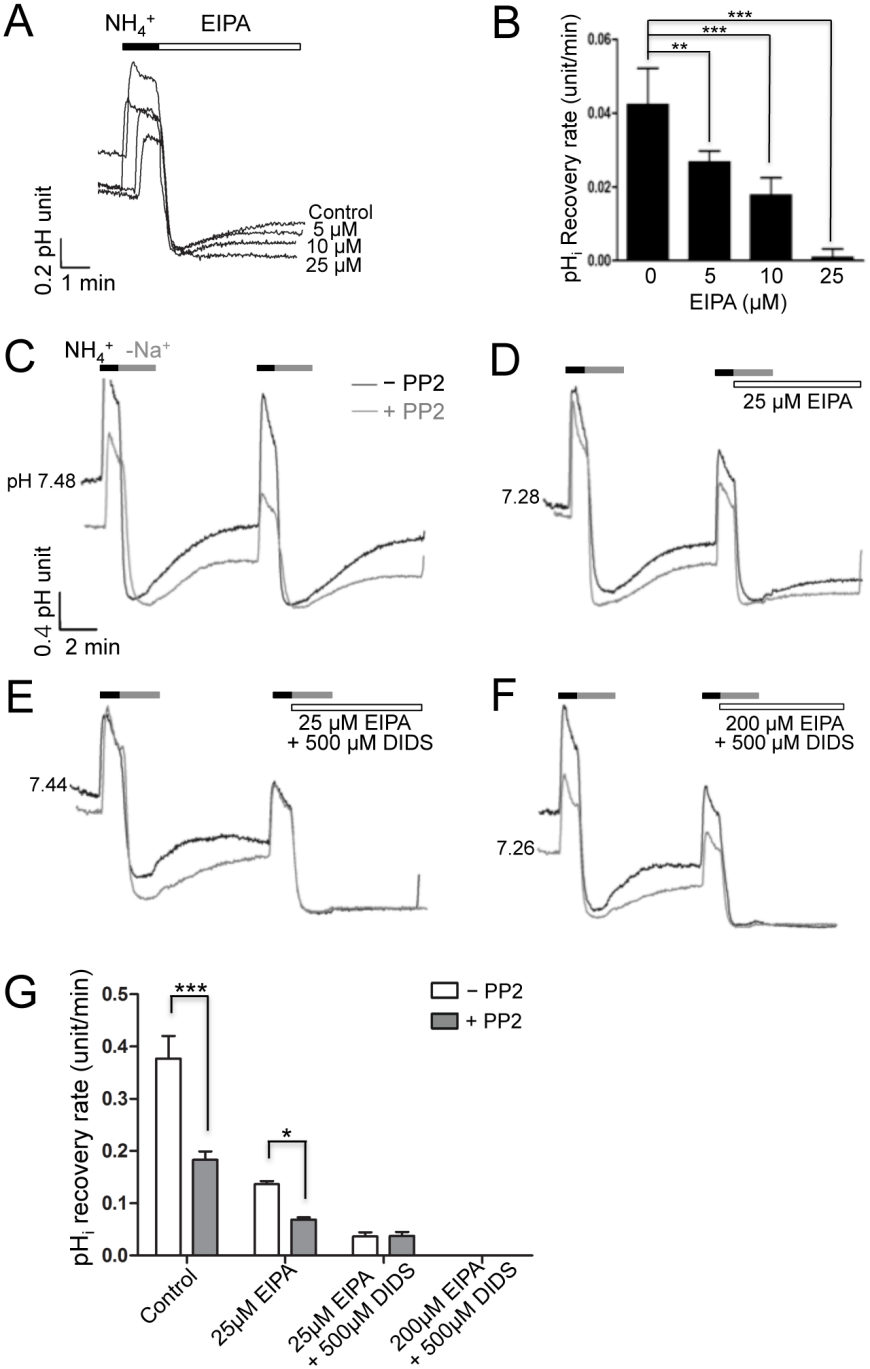
pH_i_ recovery of HSG cells is inhibited by PP2. (A and B) The pH_i_ of HSG cells in HBS was obtained in the absence or presence of 5, 10, or 25 μM EIPA and the results were summarized (C-F) pH_i_ measurements were performed using HSG cells in BBS, and the effects of pre-treatment with 5 μM PP2 for 20 min were evaluated (grey trace). (G) Summary of pH_i_ recovery rates in HSG cells. The data are presented as the mean ± S.E. (error bars) (*, P < 0.05; ***, P < 0.001)

In addition to hSMG acinar cells, we also studied the activities of NBCs in an HSG cell line. Specifically, we first confirmed the effective concentration of EIPA needed to block NHE activity in HSG cells by measuring its effect on the rate of pH_i_ recovery in a HEPES buffered solution (Fig 3A). We found that the pH_i_ recovery rate of 0.042 ± 0.004 pH units/min (n = 5) was completely inhibited by 25 μM EIPA (n = 6, Fig 3B).

We next measured pH_i_ in BBS to examine the effect of PP2 on NBCe1-B and NBCn1 activities in HSG cells using the same technique as for hSMG acinar cells. The pH_i_ recovery rate in HSG cells (0.377 ± 0.043 pH units/min, n = 9, Fig 3C, black line) was less than that of hSMG acinar cells (Fig 2C), and was decreased by ~50% upon pre-incubation with 5 μM PP2 (0.183 ± 0.016 pH units/min, n = 9, Fig 3C, grey line). When the cells were exposed to 25 μM EIPA to inhibit the NHE activity, the pH_i_ recovery rate of PP2 pretreated HSG cells (0.069 ± 0.004 pH units/min, n = 10, Fig 3D, grey line) was significantly different from control treated cells (0.136 ± 0.006 pH units/min, n = 9, Fig 3D, black line). In addition, the pH_i_ recovery by NBCe1-B and NBCn1 was inhibited by 25 μM EIPA supplemented with DIDS (0.037 ± 0.008 pH units/min, n = 7, Fig 3E) and was completely blocked by a combination of DIDS and 200 μM EIPA (n = 5, Fig 3F). The pH_i_ recovery rates of HSG cells are summarized in Fig 3G.

### Overexpression of NBCe1-B and NBCn1 in HSG cells

**Fig 4:**
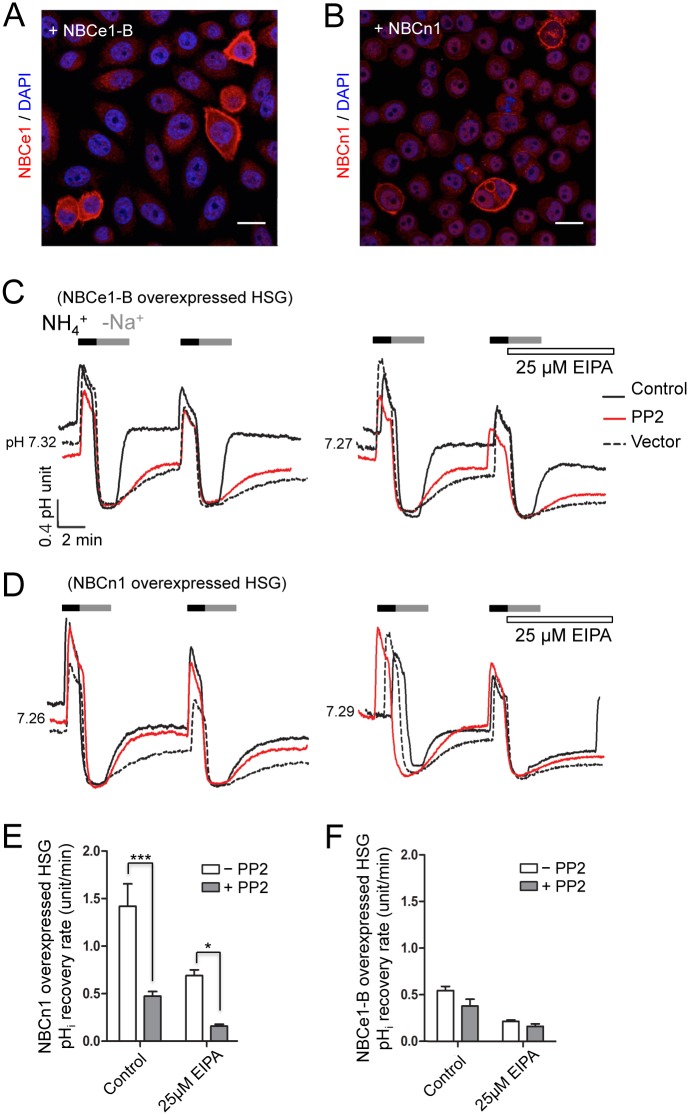
Transfected NBCe1-B is affected by PP2. (A and B) Flag-NBCe1-B and NBCn1 were transfected into HSG cells and overexpression was confirmed by immunofluorescence assay. (C and D) pH_i_ recovery rates were recorded in HSG cells overexpressing NBCe1-B or NBCn1. Horizontal bars indicate all applications. (E and F) Graphical summary of pH_i_ recovery rates. The data are presented as the mean ± S.E. (error bars) (*, P < 0.05; ***, P < 0.001).

To establish definitively whether Src kinase modulate the activity of NBCe1-B or NBCn1, HSG cells overexpressing either NBCe1-B or NBCn1 were generated and the subsequent effect on intracellular pH was measured. Overexpression of NBCe1-B and NBCn1 was confirmed via immunocytochemistry (Fig 4A and 4B). Overexpression of NBCe1-B increased the pH_i_ recovery rate (1.419 ± 0.235 pH units/min, n = 12, Fig 4C, black trace), but this was suppressed by PP2 (0.474 ± 0.048 pH units/min, n = 12, red trace). Moreover, addition of 25 μM EIPA to NBCe1-B overexpressing cells shifted the pH_i_ recovery rate to 0.690 ± 0.060 pH units/min (n = 11), which was decreased to 0.159 ± 0.021 pH units/min (n = 11) upon pre-treatment with PP2, suggesting that NBCe1-B is affected by PP2.

Overexpression of NBCn1 in HSG cells also increased the pH_i_ recovery rate (0.493 ± 0.067 pH units/min, n = 10, Fig 4D, black trace) compared with control cells (0.377 ± 0.043 pH units/min, dotted trace), indicating that NBCn1 regulates pH_i_ similar to NBCe1-B. On the other hand, the pH_i_ recovery rate induced by NBCn1 overexpression was not significantly decreased by PP2 (0.381 ± 0.073 pH units/min, n = 8, red trace). Likewise, upon incubation with 25 μM EIPA, a pH_i_ recovery rate of 0.218 ± 0.012 pH units/min (n = 3) was noted, which decreased to 0.162 ± 0.027 pH units/min (n = 4) in the presence of PP2; however, this difference was not significant, suggesting that NBCn1 is not regulated by Src kinase. The pH_i_ recovery rates of HSG cells overexpressing NBCe1-B and NBCn1 are summarized in Fig 4E and 4F. The above results were consistent with the shown in Figs 2 and 3, indicating that Src kinase regulates the activity of NBCe1-B only, and not that of NBCn1.

### NBCe1-B tyrosine residue phosphorylation and NBCe1-B translocation by Src kinase

**Fig 5:**
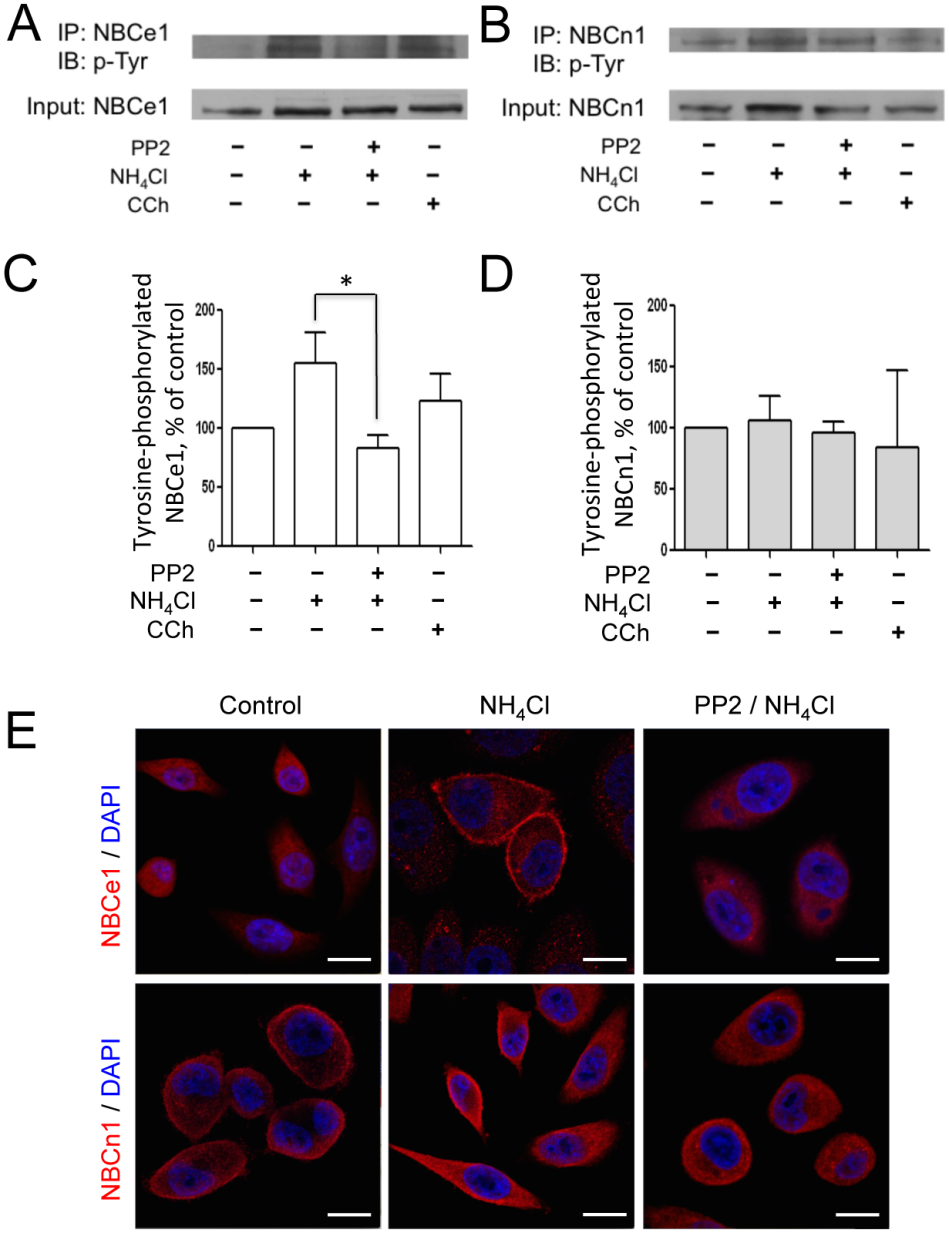
PP2 inhibits tyrosine phosphorylation and translocation of NBCe1-B, but NBCn1, in HSG cells. (A and B) Cell lysates were subjected to immunoprecipitation with NBCe1 and NBCn1 antibodies and evaluated by Western blotting with a phosphotyrosine antibody. The cells were pre-incubated in 20 mM of NH_4_Cl for 2 mins, in 5 μM of PP2 for 20 mins, and in 50 μM of CCh for 5 mins. The input control comprised 5% of the lysates. (C and D) Phosphorylated NBCe1 and NBCn1 were quantified based on protein band intensities. The data are shown as the mean ± S.E. (error bars) (n = 4; *, P < 0.05). (E) Locations of NBCe1 and NBCn1 in response of ammonium pulse in the presence or absence of PP2 were confirmed using immunocytochemistry. (Bar = 20 μm).

We next hypothesized that Src tyrosine kinase may affect NBC activity by phosphorylating tyrosine residues on NBCe1-B or NBCn1. To investigate this possibility, we performed co-immunoprecipitation assays using HSG cells. The degree of NBCe1-B tyrosine phosphorylation was significantly increased by NH_4_Cl, but was suppressed by PP2 (n = 4, Fig 5A and 5C). Contrary to NBCe1-B, tyrosine on NBCn1 was not affected either by NH_4_Cl or PP2, indicating that Src tyrosine kinase has no effect on NBCn1 phosphorylation (n = 4, Fig 5B and 5D). Carbachol was used as a positive control to stimulate NBCs.

Immunocytochemistry analysis also showed that the NBCe1-B, which was located at the plasma membrane by NH_4_Cl, remained in the cytosol when Src tyrosine kinase was inhibited by PP2 pre-treatment (Fig 5E). On the other hand, NBCn1 was constitutively expressed on the plasma membrane side. Consistent with our result thus far, these findings suggested that Src tyrosine kinase influences only the activity of NBCe1-B, and not that of NBCn1.

## Discussion

In the present study, we identified the expression and cellular localization of specific isoforms of NBCe1 and NBCn1 in human submandibular glands (hSMG) by RT-PCR and immunohistochemistry. Intracellular pH measurement and co-immunoprecipitation studies confirmed the activities of NBCs, especially NBCe1-B and NBCn1 in hSMG acinar and HSG cells. In addition, our data demonstrated that only NBCe1-B activity is regulated by Src tyrosine kinase.

We first asked which NBC isoforms are expressed in hSMGs. NBC expression was previously demonstrated in an HSG cell line originating from hSMG ducts. We confirmed that all of the NBC isoforms in hSMG ducts were expressed in an identical pattern in HSG cells. Specifically, RT-PCR and immunofluorescence studies were used to determine the isoforms of NBCe1 or NBCn1 expressed in hSMGs and HSG cells and their cellular localization. Our immunohistochemical studies demonstrated that NBCn1 was expressed at the basolateral membrane of acinar cells in hSMGs (Fig 1). Consistent with this data, NBCn1 is also expressed on the basolateral side of the ParC5 rat parotid acinar cell line [20, 21], suggesting that NBCn1 may be involved in HCO_3_^-^ influx and pH_i_ regulation in acinar cells as well as in other secretory epithelial cells [22–24], including the murine duodenum [25]. In our experiments, NBCn1 appeared to function as a major pH_i_ regulator in hSMG acinar cells. Specifically, decreased pH_i_ in hSMG acinar cells evoked by an ammonium pulse recovered rapidly to prestimulus levels, which was inhibited by high concentrations of EIPA, an inhibitor of NBCn1 [24, 26] (Fig 2). On the other hand, NBCn1, referred to as NBC3 (GeneBank accession no. 047033) in Ref. 28, was expressed on the luminal side of hSMG duct cells, which was consistent with previous work on human salivary glands [27, 28]. Indeed, apical NBCn1 likely functions as an intracellular pH regulator and HCO_3_^-^ salvage mechanism to maintain acidic saliva in human salivary glands in a resting state [26, 29].

In rat parotid glands, NBCe1-B is expressed at the basolateral membrane of acinar cells [30], and is also located at the basolateral side of human parotid acinar cells as an acid extruder for intracellular pH regulation and HCO_3_^-^ ion absorption from the basolateral side [31]. However, unlike cells of the parotid gland, NBCe1-B is expressed on the apical membrane of hSMG duct cells as shown in Fig 1B, suggesting that NBCe1-B may also function as an HCO_3_^-^ salvage mechanism in resting state cells, similar to NBCn1.

Resting saliva, in which submandibular and sublingual glands play a dominant role, contain low HCO_3_^-^ concentrations [32, 33]. NBCe1 was expressed on the apical membrane of hSMG ducts (Fig 1B), and appeared to play a salvage role by absorbing HCO_3_^-^. On the other hand, stimulated saliva in which the parotid glands play a dominant role contains high concentrations of HCO_3_^-^. Indeed, in the human parotid glands, NBCe1 is also expressed at the basolateral membrane in acinar cells [31], which may enable parotid glands to accumulate more HCO_3_^-^.

Although the specific mechanisms of NBCs activities were not fully identified in this study, several studies have investigated the signaling molecules that regulate the activities of NBCs such as Ca^2+^ [34], cAMP [35], PKA [36], and PKC [37]. In addition, a few other studies have looked into whether CO_2_-induced renal NBC activity is modulated by Src kinase [38, 39]. The activity of Src kinase is increased by CO_2_, low intracellular pH, and metabolic acidosis [40, 41]; however, there is currently no evidence regarding which specific NBC isoform is regulated by Src kinase and whether Src kinase directly phosphorylates tyrosine residues on NBCs or if it is a part of an upstream signaling cascade. Thus, in this study, we examined whether PP2, a Src kinase inhibitor, can inhibit pH_i_ recovery mediated by two different types of NBCs. Our data demonstrated that the activity of NBCe1-B is regulated by Src kinase in hSMG acinar and HSG cells, while that of NBCn1 is not. pH_i_ recovery was not affected by PP2 in hSMG acinar cells in which only NBCn1 was expressed (Fig 2C). On the other hand, when HSG cells containing both NBCe1-B and NBCn1 were exposed to PP2, the pH_i_ recovery rate was significantly decreased (Fig 3C), indicating that NBCe1-B might be modulated by Src kinase. In the presence of 25 μM EIPA, the pH_i_ recovery rate was affected by PP2 pre-treatment when both NBCe1 and NBCn1 were functionally expressed (Fig 3D). As shown in Fig 2, expression of NBCe1-B differs between hSMG acinar cells and HSG cells, in that only hSMG acinar cells express NBCn1, which is not affected by PP2. Indeed, because the HSG cell line only expresses NBCe1-B, we were able to confirm that only NBCe1-B is regulated by Src tyrosine kinase, while NBCn1 is not. The data in Fig 3C and 3D demonstrate a significant regulation of EIPA-sensitive pH_i_ recovery, which is regulated by NHE in HSG cells, suggesting this process is influenced by Src tyrosine kinase. The result was further confirmed by the overexpression of either NBCn1 or NBCe1-B in HSG cells. Specifically, the pH_i_ recovery rate in cells overexpressing NBCn1 was not changed by PP2 (Fig 4D), while overexpression of NBCe1-B led to a decreased pH_i_ recovery rate following pre-treatment with PP2 (Fig 4C). Taken together, these data indicate that only NBCe1-B is modulated by Src tyrosine kinase (Fig 4C).

**Fig 6:**
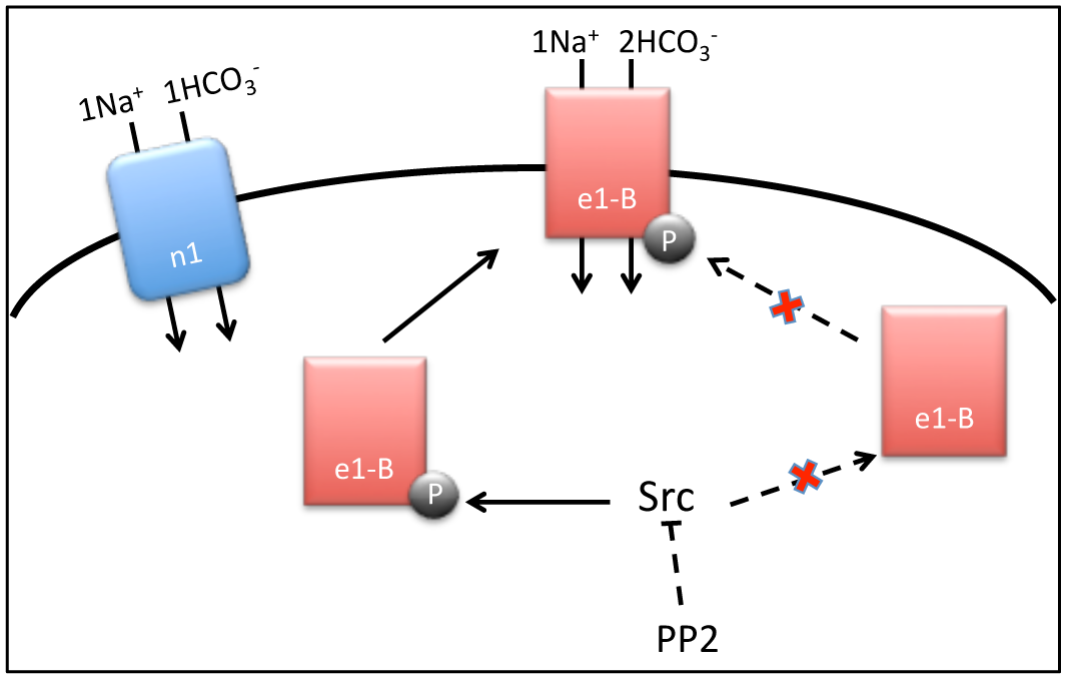
Schematic model of NBCe1-B and NBCn1 regulation by Src kinase. NBCe1-B and NBCn1 mediate intracellular pH in an HSG cell line and human submandibular glands, especially on the apical side of duct cells. NBCe1-B is phosphorylated by Src kinase and translocates to the plasma membrane, whereas the NBCn1 is not regulated by Src kinase. The effect of Src kinase is inhibited by PP2. Arrows indicate activation and bars indicate inhibition.

In the present study, we noted that phosphorylation of NBCe1 tyrosine residues by an NH_4_Cl pulse was suppressed by PP2 using co-immunoprecipitation experiments (Fig 5A), and that only phosphorylated NBCe1 was able to translocate from the cytoplasm to the plasma membrane (Fig 5E). However, NH_4_Cl did not affect the phosphorylation status of NBCn1 tyrosine residues, nor was the phospho-tyrosine of NBCn1 dephosphorylated by PP2 (Fig 5B). Carbachol (CCh) was used as a positive control in the experiments shown in Fig 5, since CCh stimulates NBCs via ERKs and a PKC-dependent pathway, which is independent of Src [42]. A schematic model summarizing the regulatory mechanisms of NBCe1-B and NBCn1 is presented in Fig 6.

It was investigated the relationship between NBC and Src kinase from several previous studies in renal cells [38, 39]. In these studies, the NBC was identified as NBCe1-A, which is known to be expressed renal cells. Moreover, these studies only showed differences in activities of NBC by Src kinase. In the present study, we not only characterized the effects of Src kinase on NBC activity, but also the phosphorylation status of tyrosine residues on activated NBCs due to Src kinase activity. In addition, we were concerned as to why NBCn1 did not associate with Src kinase, and also which molecule is responsible for NBCn1 activation. We hypothesized that another kinase may be responsible for NBCn1 activation, since it has an abundance of putative serine phosphorylation sites compared with tyrosine phosphorylation sites (analyzed by NetPhos 2.0, http://www.cbs.dtu.dk). Moreover, differences in membrane trafficking regulation of electrogenic type and electroneutral type NBC have been observed [21].

In conclusion, we confirmed that electrogenic NBCe1-B and electroneutral NBCn1 are expressed in hSMGs. We found that NBCe1 is localized to the apical membrane of duct cells while NBCn1 is expressed on the basolateral side of acinar cells and the apical side of duct cells. We also demonstrated that NBCe1-B is modulated and phosphorylated by Src kinase, whereas NBCn1 is not regulated by Src kinase. Taken together, our results suggest that Na^+^-HCO_3_^-^ cotransporters, especially NBCe1-B and NBCn1, play an important role in hSMG pH_i_ regulation and are regulated by different mechanisms. Future work will focus on the molecules that regulate NBCn1 activity and their respective mechanisms of action.

## Supporting Information

S1 Fig Intracellular pH calibration from BCECF-loaded HSG cells. (A) Fluorescence ratio (490/440 nm) changes during exposure to nigericin-containing solutions at pH 6.2, 6.6, 7.0, 7.4, and 7.8. (B) Dependence of fluorescence ratio on intracellular pH (n = 16).
(TIF)
