# Supplementary figures and images for: Role of Sodium Bicarbonate Cotransporters in Intracellular pH Regulation and Their Regulatory Mechanisms in Human Submandibular Glands
Source: PLoS One. 2015 Sep 16;10(9):e0138368. doi: 10.1371/journal.pone.0138368 (PMC4573515; doi:10.1371/journal.pone.0138368)

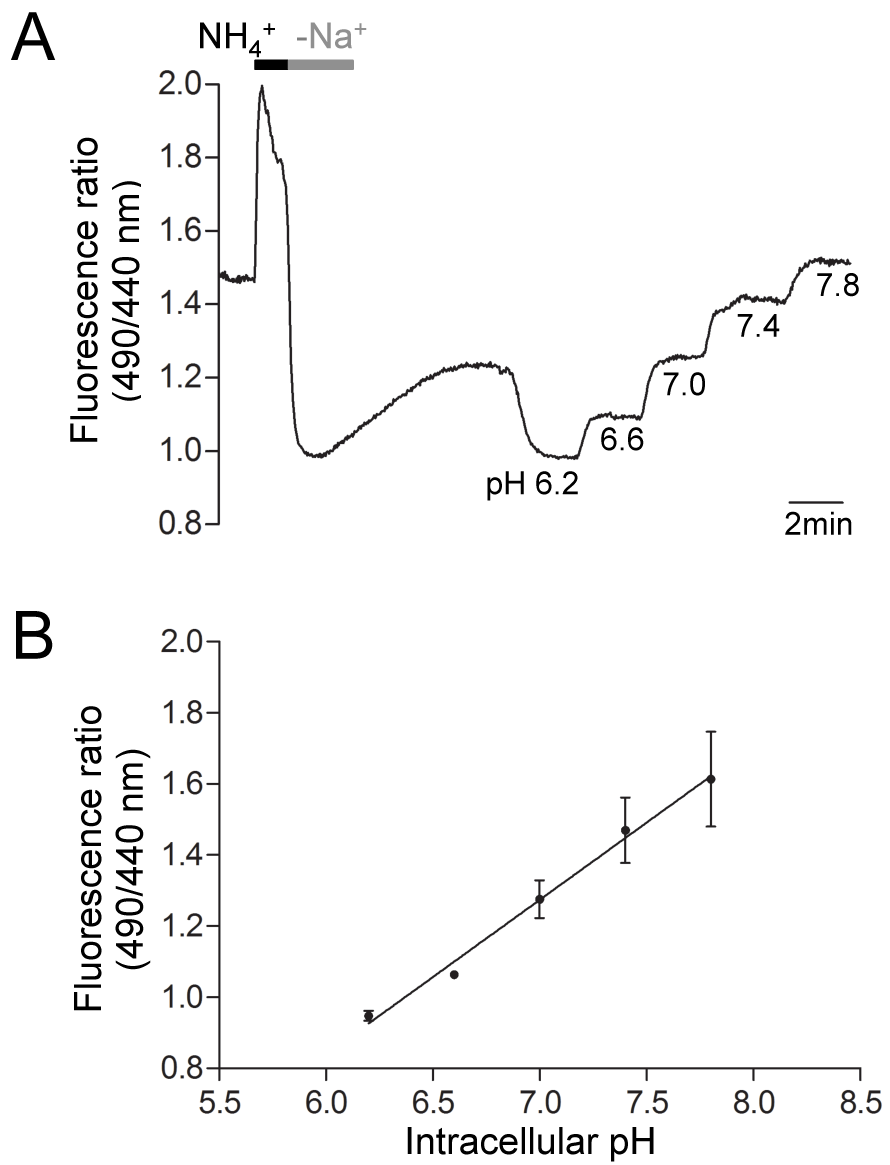

Supplement: S1 Fig — (A) Fluorescence ratio (490/440 nm) changes during exposure to nigericin-containing solutions at pH 6.2, 6.6, 7.0, 7.4, and 7.8. (B) Dependence of fluorescence ratio on intracellular pH (n = 16). (TIF) [file pone.0138368.s001.tif]
